# Excitonic Insulator Enabled Ultrasensitive Terahertz Photodetection with Efficient Low‐Energy Photon Harvesting

**DOI:** 10.1002/advs.202204580

**Published:** 2022-11-10

**Authors:** Zhuo Dong, Wanlong Guo, Libo Zhang, Yan Zhang, Jie Chen, Luyi Huang, Cheng Chen, Liu Yang, Zeqian Ren, Junrong Zhang, Wenzhi Yu, Jie Li, Lin Wang, Kai Zhang

**Affiliations:** ^1^ CAS Key Laboratory of Nanophotonic Materials and Devices & Key Laboratory of Nanodevices and Applications *i*‐Lab Suzhou Institute of Nano‐Tech and Nano‐Bionics (SINANO) Chinese Academy of Sciences Ruoshui Road 398 Suzhou Jiangsu 215123 P. R. China; ^2^ School of Nano‐Tech and Nano‐Bionics University of Science and Technology of China Jinzhai Road 96 Hefei Anhui 230026 P. R. China; ^3^ State Key Laboratory for Infrared Physics Shanghai Institute of Technical Physics Chinese Academy of Sciences 500 Yu‐tian Road Shanghai 200083 P. R. China; ^4^ Songshan Lake Materials Laboratory Dongguan Guangdong 523000 P. R. China

**Keywords:** 2D materials, exciton insulator, light–matter interaction, photodetection, terahertz

## Abstract

Despite the interest toward the terahertz (THz) rapidly increasing, the high‐efficient detection of THz photon is not widely available due to the low photoelectric conversion efficiency at this low‐energy photon regime. Excitonic insulator (EI) states in emerging materials with anomalous optical transitions and renormalized valence band dispersions render their nontrivial photoresponse, which offers the prospect of harnessing the novel EI properties for the THz detection. Here, an EI‐based photodetector is developed for efficient photoelectric conversion in the THz band. High‐quality EI material Ta_2_NiSe_5_ is synthesized and the existence of the EI state at room temperature is confirmed. The THz scanning near‐field optical microscopy experimentally reveals the strong light–matter interaction in the THz band of EI state in the Ta_2_NiSe_5_. Benefiting from the strong light–matter interaction, the Ta_2_NiSe_5_‐based photodetectors exhibit superior THz detection performances with a detection sensitivity of ≈42 pW Hz^−1/2^ and a response time of ≈1.1 µs at 0.1 THz at room temperature. This study provides a new avenue for realizing novel high‐performance THz photodetectors by exploiting the emerging EI materials.

## Graphical abstract

High‐performance terahertz (THz) photodetectors based on excitonic insulator (EI) material Ta_2_NiSe_5_ are reported. The THz scanning near‐field optical microscopy experimentally reveals the strong light‐matter interaction in the THz band of EI state in Ta_2_NiSe_5_. Benefitting from the strong interaction, the photodetectors exhibit superior THz detection performances with an ultra‐high sensitivity and fast response speed at room temperature.

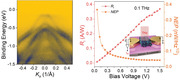

## 1 Introduction

Nowadays, high‐efficient photodetection in the long‐wave infrared especially in far infrared up to terahertz (THz) range (30 µm to 3 mm) at room temperature, is particularly rare but becomes more and more important. THz wave is located in the cross transition region of microwave electronics and infrared photonics, which promises it a wide range of applications, including security check, biomedical, wireless communication, and remote sensing.^[^ ^1^, ^2^, ^3^, ^4^, ^5^, ^6^, ^7^ ^]^ Current THz detectors based on different device structures and detection mechanisms of pyroelectric/Golay cells,^[^ ^8^ ^]^ Schottky diode detectors,^[^ ^9^ ^]^ quantum well detectors,^[^ ^10^ ^]^ bolometers,^[^ ^11^ ^]^ and field‐effect transistors detectors^[^ ^12^ ^]^ suffer from some drawbacks, such as low sensitivity, complex material and device fabrication process, or low‐temperature dependence.^[^ ^13^, ^14^, ^15^ ^]^ The main challenge and technical bottleneck of THz photodetection is the large mismatch between the nanoscale active region and the extremely long wavelength of the incident THz wave, resulting in low photoelectric conversion efficiency. In order to break through this technical bottleneck in THz detection, it is crucial to develop novel materials with strong light–matter interactions for efficient photoelectric conversion of low‐energy photons.

Quantum states in emerging materials, such as topological states, superconductivity, and unconventional excitonic states, have inspired innovations in the fields of electronic and optoelectronic devices.^[^ ^16^, ^17^, ^18^, ^19^, ^20^, ^21^, ^22^ ^]^ While, excitonic insulator (EI) states as the novel quantum states with anomalous optical transitions and renormalized valence band dispersion has gained rediscovery due to its unique band structure and accompanied photoelectric characteristics,^[^ ^23^, ^24^, ^25^ ^]^ which can be realized in narrow band gap semiconductors or small band overlapped semimetals at finite temperatures.^[^ ^26^, ^27^ ^]^ Specifically, when the exciton binding energy (*E*_b_) exceeds the band gap (*E*_g_), the excitons spontaneously generate and condense into a completely new ground state due to the weakly screened Coulomb interaction between electrons and holes.^[^ ^28^, ^29^ ^]^ However, due to the difficulty in finding suitable compound materials with an EI state, the EI state has been considered as an unconfirmed quantum state.^[^ ^30^ ^]^ In recent years, the EI state has been experimentally confirmed in a handful of strongly correlated systems (TmSe_1‐_ *_x_* Te*_x_*,^[^ ^31^ ^]^ 1T‐TiSe_2_,^[^ ^32^ ^]^ and InAs/GaSb^[^ ^33^ ^]^), which provide a unique platform for the study of quantum many‐body physics and optoelectronic devices due to their exotic properties.

Recently, 2D transition metal chalcogenide compounds (TMC) with narrow bandgap or semi‐metallicity have also shown promise as hosts of the novel EI ground state.^[^ ^34^, ^35^, ^36^ ^]^ Ta_2_NiSe_5_, a ternary layered TMC compound with a narrow direct energy gap structure has been identified as a stable EI material.^[^ ^37^, ^38^ ^]^ Over the past few years, the EI state of Ta_2_NiSe_5_ at room temperature has been widely studied and characterized.^[^ ^39^, ^40^, ^41^ ^]^ The electrical transport tests on bulk and few‐layer samples have confirmed that there is a transition from semiconductor to EI state in Ta_2_NiSe_5_ at phase transition temperature *T*_c_ ≈ 326 K.^[^ ^28^, ^30^ ^]^ At the same time, the crystal structure of Ta_2_NiSe_5_ undergoes an orthorhombic to monoclinic distortion across in the second‐order phase transition.^[^ ^42^, ^43^ ^]^ Angle‐resolved photoelectron spectroscopy (ARPES) experiments showed that the valence band edges are unusually flat while the energy band gap enlarges as the temperature decreases below the *T*_c_.^[^ ^44^, ^45^ ^]^ In addition, giant Fano resonance and pump‐probe time‐resolved measurements further revealed strong band hybridization and interband interactions.^[^ ^46^, ^47^ ^]^ The special EI state of Ta_2_NiSe_5_ provides an unprecedented opportunity to study the role of electronic states in the exciton formation on electronic and optoelectronic devices. However, the current research on Ta_2_NiSe_5_ mostly focuses on its physical properties, such as how to demonstrate and modulate the EI state,^[^ ^41^, ^48^, ^49^, ^50^ ^]^ its applications including photodetections are rarely researched.^[^ ^51^, ^52^, ^53^, ^54^ ^]^ The expected strong light–matter interaction in the EI material Ta_2_NiSe_5_ is still elusive and the harnessing of the EI properties in the THz photodetection is far unexplored.

In this work, we demonstrate a photodetector based on the EI material Ta_2_NiSe_5_ and utilize the novel EI state to achieve sensitive THz detection with highly efficient low‐energy photon harvesting. First, the high‐quality Ta_2_NiSe_5_ crystals are synthesized by the chemical vapor transport (CVT) method, and the existence of the EI state at room temperature and the second‐order phase transition at *T*_c_ ≈ 328 K are confirmed by the combination of various characterization methods. Furthermore, we reveal that the EI state of Ta_2_NiSe_5_ has strong light–matter interaction in the THz band through near‐field THz microscopy. Benefiting from the strong light–matter interaction at the THz band, the EI‐based photodetectors achieve a high sensitivity of ≈42 pW Hz^−1/2^ and a fast response speed of ≈1.1 µs at room temperature. This work opens up the possibility to explore new fundamental quantum states for the future applications in THz optoelectronic devices.

## 2 Results and Discussion

**Figure 1:**
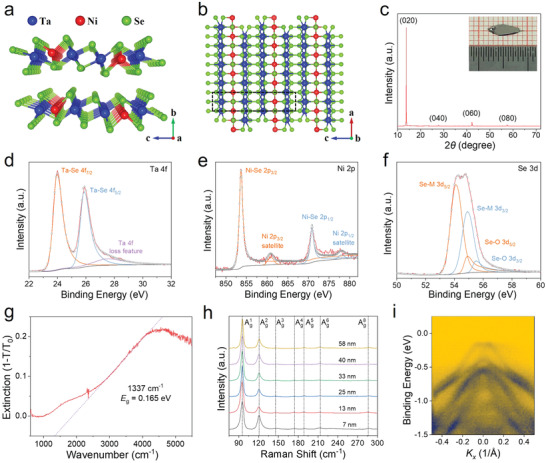
Characterizations of the Ta_2_NiSe_5_ single crystals. a) Atomic structure of the Ta_2_NiSe_5_ stacked along the *b* axis. b) Top view of crystallographic structure of the Ta_2_NiSe_5_. c) XRD pattern of the Ta_2_NiSe_5_ crystals. Inset shows the optical image of the Ta_2_NiSe_5_ single crystal. d–f) High‐resolution XPS spectra of Ta 4f, Ni 2p, and Se 3d core regions in the Ta_2_NiSe_5_ crystal, respectively. g) FTIR absorption spectrum of the mechanically exfoliated Ta_2_NiSe_5_ nanosheets. h) Raman spectra of the Ta_2_NiSe_5_ nanoflake with different thickness. i) Band structure of the Ta_2_NiSe_5_ single crystal measured by ARPES at *T* = 300 K along the $\bar{\Gamma }$–$\bar{X}$ direction.

The ternary compound Ta_2_NiSe_5_ is a layered structure stacked by a weak van der Waals force along the *b*‐axis, which possesses a monoclinic atomic structure at room temperature. As shown in **Figure** **1**a, each layer is a sandwich‐like structure, where the top and bottom Se atoms sandwich the middle Ta/Ni atoms running along the *c* axis. The top view of the crystallographic structure of Ta_2_NiSe_5_ is depicted in Figure 1b, in which the [TaSe_6_]_2_ dimer chains and NiSe_4_ single chains are arranged periodically along the *c*‐axis and run along the *a*‐axis to form the quasi‐one‐dimensional structure. To investigate the crystallographic structure of our synthesized Ta_2_NiSe_5_, X‐ray diffraction (XRD) pattern was performed, as displayed in Figure 1c. The four diffraction peaks at 13.8°, 27.8°, 42.2°, and 57.4° are assigned to the (020), (040), (060), and (080), respectively, and can be matched well with the monoclinic space group C2/*c* (PDF#78‐0279), which is consistent with the EI phase structure. Meanwhile, these strong peaks illustrate the high‐purity phase and highly preferred orientation along the (0*l*0) direction of Ta_2_NiSe_5_ single crystal. Furthermore, the scanning electron microscopy (SEM) with energy dispersive X‐ray spectroscopy (EDX) was adopted to characterize the surface morphology and elemental composition of Ta_2_NiSe_5_, as shown in Figure S1, Supporting Information. The bulk crystal shows a typical layered structure with uniform distribution of Ta, Ni, and Se elements, and the atomic ratio is about 2:1:5. In the meanwhile, X‐ray photoelectron spectroscopy (XPS) was employed to characterize the chemical state and chemical composition of the Ta_2_NiSe_5_ crystal. As depicted in Figure S2, Supporting Information, The wide‐scan XPS spectrum clearly shows the signals of Ta, Ni, and Se elements. Figure 1d–f shows the fitted high‐resolution XPS spectra of Ta 4f, Se 3d, and Ni 2p core regions, respectively. The peaks located around 24 eV and 25.9 eV in Figure 1d can be identified as Ta 4f_7/2_ and Ta 4f_5/2_ of the Ta–Se bonding. Figure 1e displays the fitted peaks at 853.6 eV and 870.9 eV, which can be indexed to the Ni 2p_3/2_ and Ni 2p_1/2_ of the Ni–Se bonding. In addition, two peaks at 54.1 eV and 54.9 eV of Se 3d spectrum displayed in Figure 1f represent the binding energies of Se^2−^ of Ta_2_NiSe_5_. Furthermore, the atomic ratio Ta, Ni, and Se was calculated to be around 1.95:1:5.05 from the XPS spectrum, which is also close to the ideal composition of Ta_2_NiSe_5_.

Moreover, the Fourier transfer infrared (FTIR) spectrometer was deployed to investigate the optical band gap of Ta_2_NiSe_5_. Figure 1g displays the infrared absorption spectrum of the mechanically exfoliated Ta_2_NiSe_5_ nanosheets on the gold substrate. The Ta_2_NiSe_5_ shows an absorption edge of ≈1337 cm^−1^, corresponding to a narrow band gap of ≈0.165 eV, which is consistent with the previous reports.^[^ ^28^ ^]^ The infrared absorption spectrum is a reproducible result for the other sample (as shown in Figure S3, Supporting Information). Figure 1h shows the Raman spectra of the mechanically exfoliated Ta_2_NiSe_5_ nanoflake with thicknesses ranging from 7 to 58 nm. The atomic force microscopy (AFM) images of the Ta_2_NiSe_5_ nanoflake are depicted in Figure S4a, Supporting Information. It can be seen that all samples exhibit seven Raman peaks, all of which are A_g_ vibration mode, which matches well with the previous reports.^[^ ^30^, ^53^ ^]^ Among them, two strong Raman peaks at 97 cm^−1^ and 122 cm^−1^ are the characteristic peaks of monoclinic structure (EI state) without obvious shift in all samples (as shown in Figure S4b, Supporting Information), illustrating the existence of EI state in the Ta_2_NiSe_5_ with different thickness at room temperature.^[^ ^30^ ^]^ With the temperature increasing to 350 K, the seven vibration peaks were also observed at the Raman spectra, as shown in Figure S5, Supporting Information. It should be noted that two characteristic Raman peaks at 97 cm^−1^ and 122 cm^−1^ have an obvious red shift, which indicates the change of EI state.^[^ ^30^ ^]^ Flattening of the valence band top is important evidence for the existence of the EI state in Ta_2_NiSe_5_, which was demonstrated by the ARPES, as depicted in Figure 1i and Figure S6, Supporting Information. The band structure in the vicinity of the Fermi level *E*_F_ in Figure 1i clearly shows the flattening of the valence band top, which is related to the opening of band gap due to the many‐body interaction in the formation of the EI state.^[^ ^44^, ^45^ ^]^ These results are direct evidence for the existence of EI state in our synthesized Ta_2_NiSe_5_ at room temperature, and prove that it is a high‐quality single crystal.

**Figure 2:**
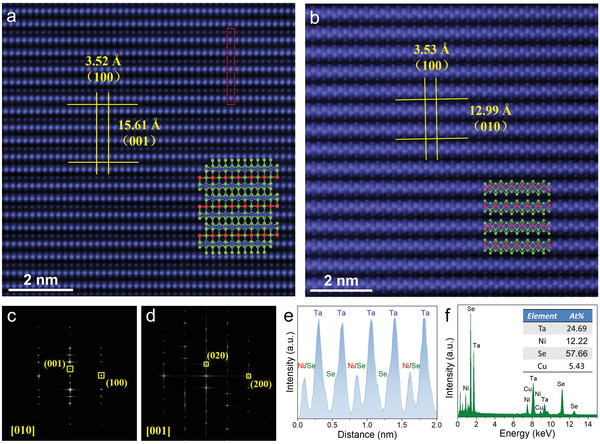
Microscopic atomic structure of the synthesized Ta_2_NiSe_5_. a,b) Top surface and cross‐section High‐magnification STEM image of the Ta_2_NiSe_5_ nanosheet (zone axis = [010] and [001]), respectively. Inset is the atomic structure diagram of Ta_2_NiSe_5_. c,d) Corresponding FFT diffraction pattern of top surface and cross‐section STEM image. e) Intensity line profile of the atomic image for red rectangle dashed box in (a). f) EDX spectroscopy of the Ta_2_NiSe_5_ nanosheet. Inset is the atomic ratio of Ta, Ni, and Se.

To further evaluate the crystalline quality, atomic structure and chemical composition of our synthesized Ta_2_NiSe_5_ were examined by the spherical‐aberration corrected scanning transmission electron microscopy (STEM). **Figure** **2**a,b displays the high‐resolution top surface and cross‐section STEM images with no obvious defects or impurities, in which each atom can be clearly distinguished, demonstrating the high crystalline quality of our synthesized Ta_2_NiSe_5_ crystal. In addition, the cross‐section STEM image (Figure 2b) shows a clear lamellar structure along the *b*‐axis. In the meanwhile, as shown in the insets of Figures 2a and 2b, the measured atomic structure matches well with the monoclinic atomic structure diagram. Furthermore, the corresponding fast Fourier transform (FFT) diffraction patterns oriented along the [010] and [001] zone axis are displayed in Figures 2c and 2d, respectively, both of which show only one set of the sharp and well‐arranged diffraction spots, further illustrating the high crystalline quality and the single‐crystal nature of Ta_2_NiSe_5_. The measured interplanar spacings of ≈3.52 Å, ≈15.61 Å, and ≈12.99 Å correspond to the (100), (001), and (010) planes of Ta_2_NiSe_5_ crystal, respectively. All of the interplanar spacings can be well matched to the monoclinic crystal structure of Ta_2_NiSe_5_, which further illustrates the existence of EI state at room temperature. The periodic arrangement of Ta, Ni, and Se atoms can be seen from the atomic intensity line profile shown in Figure 2e. It should be noted that the Ni and Se atoms are completely coincident at some sites and cannot be distinguished accurately. Figure 2f displays the EDX spectroscopy of Ta_2_NiSe_5_ nanoflake, which shows that the atomic ratio of as‐prepared Ta_2_NiSe_5_ is close to 2:1:5. The low‐magnification high‐angle annular dark‐field (HAADF) STEM image and element mapping images are depicted in Figure S7a, Supporting Information, confirming the uniform element distribution of the Ta_2_NiSe_5_. In the meanwhile, the uniform element distribution is also shown in the cross‐section element mapping images of Ta_2_NiSe_5_, as depicted in Figure S7b, Supporting Information. The above results suggest the high crystalline quality and stable EI state of the synthesized Ta_2_NiSe_5_ crystals at room temperature, which plays an important role in the subsequent THz photodetections.

Subsequently, the Ta_2_NiSe_5_‐based field‐effect transistors (FET) and Hall bar devices were fabricated on the degenerately p‐type doped silicon (Si) substrate (more details see Experimental Section) to study the electrical properties of Ta_2_NiSe_5_. Figure S8a, Supporting Information, shows the output characteristic curve of the Ta_2_NiSe_5_ FET, which displays a weak gate‐control ability and a good ohmic contact between Ta_2_NiSe_5_ nanosheet and the metal electrodes. As displayed in Figure S8b, Supporting Information, the transfer characteristics for the Ta_2_NiSe_5_ FET exhibit a typical n‐type semiconductor characteristic. The temperature dependence of the longitudinal resistance *R*_XX_ was performed from 50 to 200 K at *B* = 0 T to understand the transport properties of the Ta_2_NiSe_5_, as shown in Figure S9a, Supporting Information. It can be seen that *R*_XX_ increases with the decreasing temperature, exhibiting typical semiconductor behavior. In the meanwhile, Figure S9b, Supporting Information, shows the resistance *R* as a function of temperature *T* from 310 to 342 K. The resistance *R* displays an anomaly at *T* ≈ 327 K, corresponding to the second‐order phase transition, which illustrates the existence of an EI state in Ta_2_NiSe_5_ below the characteristic temperature *T*_c_ ≈ 327 K. Below *T*_c_, the rapid increase in resistance is related to the opening of band gap under the EI state. These results are consistent with the previous reports.^[^ ^28^, ^30^ ^]^

**Figure 3:**
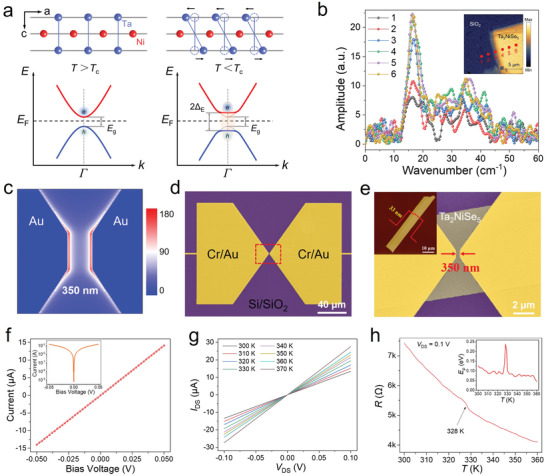
Strong light–matter interaction and electrical properties of the Ta_2_NiSe_5_ photodetectors. a) Schematic diagram of the lattice distortion (upper panel) and the band structure (bottom panel) of Ta_2_NiSe_5_ crystals during the formation of EI state. b) Normalized THz near‐field signal spectra of the Ta_2_NiSe_5_ nanoflake at different positions. Inset is the THz near‐field microscopy (2nd order signal) mapping image. c) Electric field distribution in the bow‐tie antenna under 0.1 THz illumination. d,e) Full‐scale false color SEM image of the Ta_2_NiSe_5_‐based THz photodetector. Inset displays the AFM image of the Ta_2_NiSe_5_ nanoflake. f) *I*–*V* curve of the Ta_2_NiSe_5_ photodetector without gate voltage. Inset is the *I*–*V* curve in logarithmic scale. g) *I*–*V* curves of the Ta_2_NiSe_5_ device at different temperature. h) Resistance of the Ta_2_NiSe_5_ device as a function of temperature at *V*_DS_ = 0.1 V. Inset shows the temperature dependence of the activation energy *E*_*ρ*_.

Considering the spontaneous crystal structure symmetry breaking and exotic valence band flattening of the EI state in Ta_2_NiSe_5_, it may bring about strong light–matter interaction. The lattice structure and band structure of the EI state in Ta_2_NiSe_5_ were depicted in **Figure** **3**a. Before the EI transition, Ta_2_NiSe_5_ crystal exhibits a standard orthogonal crystal structure and a narrow gap semiconductor band structure. During the formation of EI states below *T*_c_, the lattice distortion of the Ta/Ni chain leads to the structure transition from orthorhombic to monoclinic, resulting in a symmetry breaking, which benefits for enhancing the light–matter interaction in THz band.^[^ ^55^ ^]^ In addition, a large number of excitons condensation between the valence and conduction bands results in the opening of the band gap and the flattening of the valence band. Meanwhile, there is a strong interaction between excitons and phonons in the special EI state due to the coherent phonon excitation, which has a resonant absorption in the THz band.^[^ ^47^, ^56^ ^]^ Furthermore, the THz time‐domain spectroscopy (TDS)‐based scattering‐type scanning near‐field optical microscope (THz s‐SNOM) system was adopted to demonstrate the interaction of the Ta_2_NiSe_5_ sample with incident THz photons. Figure S10a, Supporting Information, shows the schematic diagram of the THz s‐SNOM experimental setup. The THz near‐field microscopy (2–4th order signals) mapping images of Ta_2_NiSe_5_ on Si/SiO_2_ substrate are displayed in Figure S10b, Supporting Information. Compared to the Si/SiO_2_ substrate, the Ta_2_NiSe_5_ nanoflake has an obvious and stronger THz field distribution in all mapping images. As depicted in Figure 3b, there is considerable optical contrast between the Ta_2_NiSe_5_ nanoflake (marked as 4–6) and the Si/SiO_2_ substrate (marked as 1–2) over the THz frequency range. These results illustrate the strong light–matter interaction in the THz band of EI state in the Ta_2_NiSe_5_ at room temperature, which can couple more THz radiation with high efficient harvesting of the low‐energy photons, and produce an excellent THz response.

In view of the strong light–matter interaction in the THz band of EI state in Ta_2_NiSe_5_, the THz photodetector was designed as a sub‐wavelength planer metal‐Ta_2_NiSe_5_‐metal (MTM) structure with a symmetric bow‐tie antenna, which can convert the incident THz radiation into the localized oscillating electric field (as shown in Figure 3c). To study the THz response performances, the Ta_2_NiSe_5_ nanoflake with a thickness of ≈33 nm (as shown in the inset in Figure 3e) was selected to fabricate the THz photodetector on the high‐resistance intrinsic Si substrate (more details see Experimental Section). Figure 3d,e displays the false‐color SEM images of the THz detector, showing a symmetric geometry with a channel length of ≈350 nm. The electrical properties of the detector was tested prior to the THz response testing. The linear *I*–*V* curve of the photodetector is shown in Figure 3f, indicating the good ohmic characteristic. Figure 3g depicts the *I*–*V* curves of the Ta_2_NiSe_5_ device at different temperature from 300 to 370 K with a step of 10 K, all of which display a nearly linear behavior, further indicating good ohmic contact. In addition, the temperature dependence of the resistance *R* of the device at source‐drain bias voltage *V*_DS_ = 0.1 V was also performed, as depicted in Figure 3h. The curve displays a weak but clear kink at *T*_c_ ≈ 328 K, demonstrating that there is also an EI phase transition. Furthermore, the activation energy *E*_*ρ*_ = −*k*_B_ *T* ^2^(∂ln*ρ*/∂*T*) was calculated from the *R*–*T* curve (as shown in the inset in Figure 3h), which is instructive for the EI transition. As can be seen, *E*_*ρ*_ exhibits a distinct jump at *T*_c_, which is consistent with previous reports.^[^ ^28^ ^]^ It further proves the existence of EI state in Ta_2_NiSe_5_ at room temperature.

**Figure 4:**
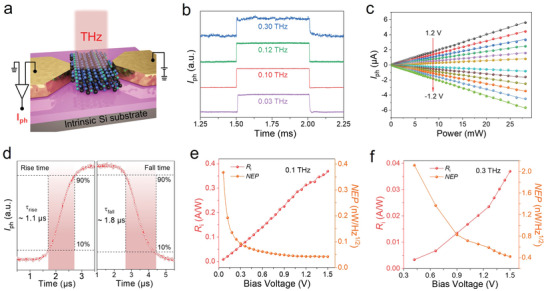
THz response properties of the Ta_2_NiSe_5_ photodetectors at room temperature. a) Schematic illustration of the Ta_2_NiSe_5_ photodetector with electrical configuration under THz irradiation. b) Time‐resolved photoresponse signal of the photodetector under 0.03, 0.10, 0.12, and 0.30 THz illumination at bias voltage of 0.1 V. c) *I*_ph_ at the frequency of 0.03 THz as a function of the incident power at different bias voltage from −1.2 V to 1.2 V with a step of 0.2 V. d) Response/recovery times for the photodetector at 0.1 THz with a bias voltage of 0.1 V. e,f) *R*_i_ and NEP of the photodetector with the function of bias voltage at 0.1 and 0.3 THz. The left axis is the *R*_i_ and right axis is the NEP.

Next, the room‐temperature THz response performances of the Ta_2_NiSe_5_ photodetector were systematically studied using a THz test system. **Figure** **4**a shows the schematic illustration of the Ta_2_NiSe_5_‐based THz photodetector with electrical configuration under THz irradiation. The THz photocurrent signals were collected by a lock‐in technique and the THz radiation was obtained from a microwave source equipped with the frequency multiplier, operating in the 0.02–0.30 THz range (for more details see Experimental Section). As shown in Figure S11, Supporting Information, the photodetector demonstrates a prominent photoresponse in the frequency of 0.07–0.12 THz and 0.24–0.30 THz, suggesting the nature of broadband detection. Furthermore, Figure 4b displays the pulsed photoresponse signal under different incident radiation frequencies. The detailed time‐resolved photoresponse spectra of the Ta_2_NiSe_5_ photodetector at different incident radiation (0.03, 0.10, 0.12, and 0.30 THz) are also depicted in Figure S12, Supporting Information. Notably, the fast and stable response waveform can be well maintained in the frequency from 0.03 to 0.30 THz, further indicating the broadband detection characteristics. As shown in Figure S13a, Supporting Information, under 0.1 and 0.3 THz illumination, *I*_ph_ of the photodetector grows approximately linearly with increasing bias voltage, which might be driven by the photoconductive effect. Figure S13b, Supporting Information, displays the *I*–*V* curve of the photodetector under pulse modulated radiation at 0.03 THz, which also shows the same growing trends. It should be noted that the *I*_ph_ at 0.3 THz is small due to the small incident power. Figure 4c exhibits the excellent linear dependence of *I*_ph_ versus incident radiation power at different bias voltages, indicating the large dynamic range and second‐order nonlinear behavior of the photodetector. The response/recovery time is defined as the time for the *I*_ph_ increases/decreases from 10%/90% of the maximum *I*_ph_ to 90%/10% at single impulse, respectively, which is recorded by a fast oscilloscope. Figure 4d displays the rise and fall times for the photodetector illuminated with 0.1 THz wave are ≈1.1 µs and ≈1.8 µs, respectively. In the meanwhile, the response/recovery time of the photodetector under 0.03, 0.12, and 0.30 THz illumination were also measured as ≈1.2 µs/≈6.9 µs, ≈1.2 µs/≈1.8 µs, and ≈1.8 µs/≈6.0 µs, respectively, as shown in Figure S14, Supporting Information.

The photocurrent responsivity (*R*_i_) and noise‐equivalent power (NEP) of the photodetector at 0.1 and 0.3 THz were calculated from the bias‐dependent *I*_ph_ to further evaluate the THz photoresponse performances, as shown in Figure 4e,f. Among them, *R*_i_ is calculated by the relation *R*_i_ = *I*_ph_/*P* (for more details see Experimental Section). It can be seen that *R*_i_ grows with increasing bias voltage for all incident THz radiation. When the bias voltage reaches 1.5 V, the values of *R*_i_ are ≈0.36 A W^−1^ and ≈0.036 A W^−1^ at 0.1 and 0.3 THz, respectively. As another important parameter, NEP, can be obtained from the formula NEP = *i*_n_/*R*_i_, where *i*_n_ is the noise current density for the photodetector. In general, the dominant noise in our test system contains the Johnson−Nyquist noise (*v*_t_), shot noise (*v*_b_), and flick noise (1/*f* noise). Among them, the 1/*f* noise is related to the change of electronic state, which usually occurs at low modulation frequency (below 1 kHz).^[^ ^5^ ^]^ During our test, the modulation frequency is over 1 kHz, so it can be ignored in our system. Therefore, the main noise sources are *v*_t_ and *v*_b_ in our system, which originate from the thermal motion of carriers due to the nonzero ohmic resistance and the generation of carriers under bias voltage,^[^ ^5^ ^]^ respectively. Inherently, the total noise current density can be obtained from the electrical characteristic of the detector by the formula *i*_n_ = (*v*_t_^2^ + *v*_b_^2^)^1/2^/*r* = (4*k*_B_ *T*/*r* + 2*qI*_DS_),^[^ ^57^ ^]^ where *k*_B_ is Boltzmann constant, *T* is the temperature, *r* is the resistance of the detector, *q* is elementary charge, and *I*_DS_ is the dark current of the detector. Figure 4e,f shows the minimum NEP of ≈42 pW Hz^−1/2^ at 0.1 THz and ≈417 pW Hz^−1/2^ at 0.3 THz, respectively. To better evaluate the performance of the Ta_2_NiSe_5_ photodetectors, a comparative survey of recently reported room‐temperature THz detectors based on different 2D materials was carried out, as displayed in **Table** **1**. It can be seen that our Ta_2_NiSe_5_ photodetector has obvious advantages in sensitivity and response speed.

**Table 1:** Performance comparison of the reported 2D materials THz detectors

Material	Frequency [THz]	Responsivity	NEP [W Hz^−1/2^]	Response time	Ref.
Graphene	0.14	450 V W^−1^	1 × 10^−10^	≈2 µs	[58]
Graphene	0.3	0.15 V W^−1^	3 × 10^−8^	—	[6]
BP	0.15	300 V W^−1^	1 × 10^−9^	≈4 µs	[59]
BP	0.29	1.7 V W^−1^	1 × 10^−10^	—	[60]
Bi_2_Te_(3−_ *_x_*_)_Se*_x_*	0.29	3 V W^−1^	1 × 10^−8^	—	[61]
MoSe_2_	0.29	38 mV W^−1^	6.6 × 10^−6^	—	[62]
SnSe_2_	0.12	170 V W^−1^	2 × 10^−10^	≈2.2 µs	[63]
PdSe_2_	0.1	20 mA W^−1^	1.42 × 10^−10^	≈7.5 µs	[64]
PtTe_2_	0.12	1.6 A W^−1^ (101 V W^−1^)	—	≈17 µs	[7]
Ta_2_NiSe_5_	0.1 (0.3)	0.36 A W^−1^ (755 V W^−1^) 0.036 A W^−1^ (75 V W^−1^)	4.2 × 10^−11^ 4.17 × 10^−10^	≈1.1 µs ≈1.8 µs	This work

## 3 Conclusion

In summary, we synthesized high‐quality Ta_2_NiSe_5_ single crystals and exploited their novel EI state for THz detection. The existence of the EI state at room temperature and the second‐order phase transition at *T*_c_ ≈ 328 K were confirmed by XRD, Raman spectroscopy, ARPES, STEM, and electrical transport tests. The EI state with spontaneous crystal structure symmetry breaking and exotic valence band flattening exhibits strong light–matter interaction in the THz band, which was underpinned by strong THz field distribution on the Ta_2_NiSe_5_ surface illustrated by the THz s‐SNOM. Based on the local electric field of the antenna‐integrated THz photodetector enhancement in MTM structure and the strong interaction of incident THz radiation and EI state, the Ta_2_NiSe_5_‐based photodetectors display a wide‐band (0.03–0.30 THz) response with a responsivity of ≈0.36 A W^−1^, an NEP of ≈42 pW Hz^−1/2^, and a response time of ≈1.1 µs at room temperature. Considering the high sensitivity and fast response speed of our Ta_2_NiSe_5_ photodetectors, this work lays the groundwork for future research into the EIs for THz photodetections.

## 4 Experimental Section

### Material Synthesis and Characterization

The high‐quality Ta_2_NiSe_5_ bulk crystals were obtained by the CVT method, the same as that of the authors’ previously reported work.^[^ ^54^ ^]^ The XRD (Bruker D8 Advance) was adopted to characterize the crystal structure of the synthesized Ta_2_NiSe_5_. The infrared absorption spectroscopy was taken from FTIR spectrometer (Bruker Vertex 70) equipped with a microscope system. A micro‐Raman system (LABRAM HR) with a 532 nm laser was performed to obtain the Raman spectrum. The morphology and thickness of the nanoflakes were characterized by optical microscope (Nikon Eclipse LV100ND) and AFM (Bruker, Dimension ICON). The atomic structure, chemical composition, and elemental mapping of Ta_2_NiSe_5_ were characterized by the STEM (FEI Titan Themis Z), SEM (Quanta FEG 250), and XPS (PHI 5000 VersaProbeIII). The energy band structure of Ta_2_NiSe_5_ single crystal was measured on the ARPES system (DA30L). The incident photon energy, energy resolution, and angular resolution were ≈21.2 eV, ≈1.8 meV, and ≈0.1°, respectively.

### Device Fabrication and Measurements

The Ta_2_NiSe_5_ nanoflakes on the Si/SiO_2_ substrate were obtained from as‐synthesized bulk crystals by the mechanical exfoliation method. It should be noted that the substrate used in the THz detectors was high‐resistance (*ρ* ≈ 20 000 Ω cm) intrinsic silicon. Subsequently, the 2‐terminal configuration, planer bow‐tie, and Hall bar electrode structures were determined by electron‐beam lithography (EBL, JEOL JBX 5500). Then, Cr/Au (10/70 nm) films were deposited by an electron‐beam evaporator (EBE, Ulvac Ei‐5Z) system, followed by a lift‐off process to form the THz photodetectors and Hall devices. The structure of THz detector with a bow‐tie antenna was characterized by the SEM. The electrical transport properties of Ta_2_NiSe_5_ devices were measured in a semiconductor parameter analyzer (Keithley 4200) and a physical property measurement System (PPMS, Quantum Design).

### THz Photoresponse Characterization

In this work, the photocurrent mode was adopted to measure the THz response measurements, in which the source electrode was grounded and the photocurrent (*I*_ph_) signal was collected at the drain electrode by means of lock‐in amplifier technique. A microwave signal source (Agilent E8257D, 0.02–0.04 THz) with different VDI frequency multiplier was selected to the THz emission source, which could tune up to 0.07–0.12 THz (WR 9 Tripler) and 0.24–0.30 THz (WR 2.8 Tripler). The THz incident radiation power intensity of 0.1 and 0.3 THz measured by the TK100 power meter were 8.6 and 10 µW mm^−2^, respectively. The photocurrent responsivity *R*_i_ of the detector could be retrieved from the formula *R*_i_ = *I*_ph_/*P*_in_×*S*_d_, where *P*_in_ and *S*_d_ is the incident radiation power intensity and the effective area of detector, respectively. The whole area of device *S*_d_ (*S*_d_ = 200 µm × 140 µm = 28 000 µm^2^ = 2.8 × 10^−2^ mm^2^) was much smaller than the diffraction‐limited area *S_*λ*_* = *λ* ^2^/4 (*S_*λ*_* for 0.1 and 0.3 THz were 2.25 and 0.25 mm^2^, respectively). So *S*_d_ was generally taken as *S_*λ*_* in the previous reports.

## Conflict of Interest

The authors declare no conflict of interest.

## Supporting information

Supporting Information

## Data Availability

The data that support the findings of this study are available from the corresponding author upon reasonable request.
