# Supplementary material for: Excitonic Insulator Enabled Ultrasensitive Terahertz Photodetection with Efficient Low‐Energy Photon Harvesting
Source: Adv Sci (Weinh). 2022 Nov 10;9(36):2204580. doi: 10.1002/advs.202204580 (PMC9798984; doi:10.1002/advs.202204580)
Supplement: Supplementary file 1 — Supporting Information [file ADVS-9-2204580-s001.pdf]

ADVANCED SCIENCE
Open Access

Supporting Information

for *Adv. Sci.*, DOI 10.1002/advs.202204580

Excitonic Insulator Enabled Ultrasensitive Terahertz Photodetection with Efficient Low-Energy Photon Harvesting

*Zhuo Dong, Wanlong Guo, Libo Zhang, Yan Zhang, Jie Chen, Luyi Huang, Cheng Chen, Liu Yang, Zeqian Ren, Junrong Zhang, Wenzhi Yu, Jie Li, Lin Wang and Kai Zhang**

Supporting Information

Excitonic Insulator Enabled Ultrasensitive Terahertz Photodetection with Efficient Low-Energy Photon Harvesting

*Zhuo Dong, Wanlong Guo, Libo Zhang, Yan Zhang, Jie Chen, Luyi Huang, Cheng Chen, Liu Yang, Zeqian Ren, Junrong Zhang, Wenzhi Yu, Jie Li, Lin Wang, Kai Zhang**

Dr. Z. Dong, Y. Zhang, J. Chen, Dr. L. Huang, C. Chen, L. Yang, Z. Ren, J. Zhang, Dr. J. Li, Prof. K. Zhang
CAS Key Laboratory of Nanophotonic Materials and Devices & Key Laboratory of Nanodevices and Applications, *i*-Lab
Suzhou Institute of Nano-Tech and Nano-Bionics (SINANO)
Chinese Academy of Sciences
Ruoshui Road 398, Suzhou, Jiangsu 215123, P. R. China
E-mail: kzhang2015@sinano.ac.cn

Dr. Z. Dong, Y. Zhang, C. Chen, L. Yang, J. Zhang
School of Nano-Tech and Nano-Bionics
University of Science and Technology of China
Jinzhai Road 96, Hefei, Anhui 230026, P. R. China

Dr. W. Guo, Dr. L. Zhang, Prof. L. Wang
State Key Laboratory for Infrared Physics
Shanghai Institute of Technical Physics
Chinese Academy of Sciences
500 Yu-tian Road, Shanghai 200083, China

Dr. W. Yu
Songshan Lake Materials Laboratory
Dongguan, Guangdong, 523000, P.R. China

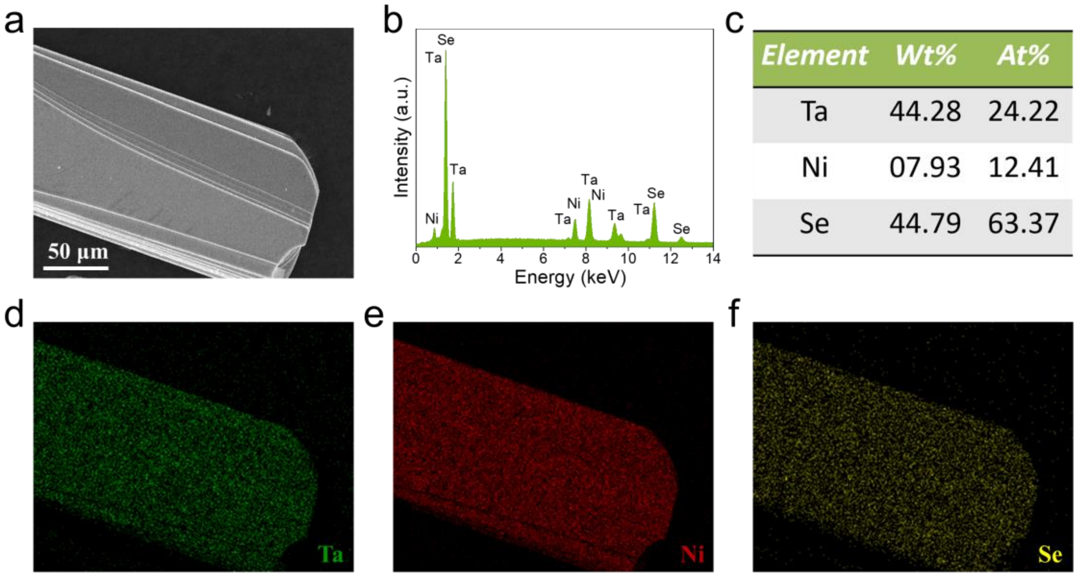

Figure S1. Morphology and chemical composition of the synthesized Ta_2NiSe_5 crystals. (a) SEM image of a Ta_2NiSe_5 crystal with characteristic layered structure. (b) EDX spectroscopy of the crystal in (a), demonstrating the existence of Ta, Ni, and Se element. (c) The atomic ratio of Ta/Ni/Se elements for the Ta_2NiSe_5. The atomic ratio of Ta:Ni:Se is approximately 2:1:5. (d-f) Corresponding EDX elemental mapping images of Ta, Ni, and Se, respectively.

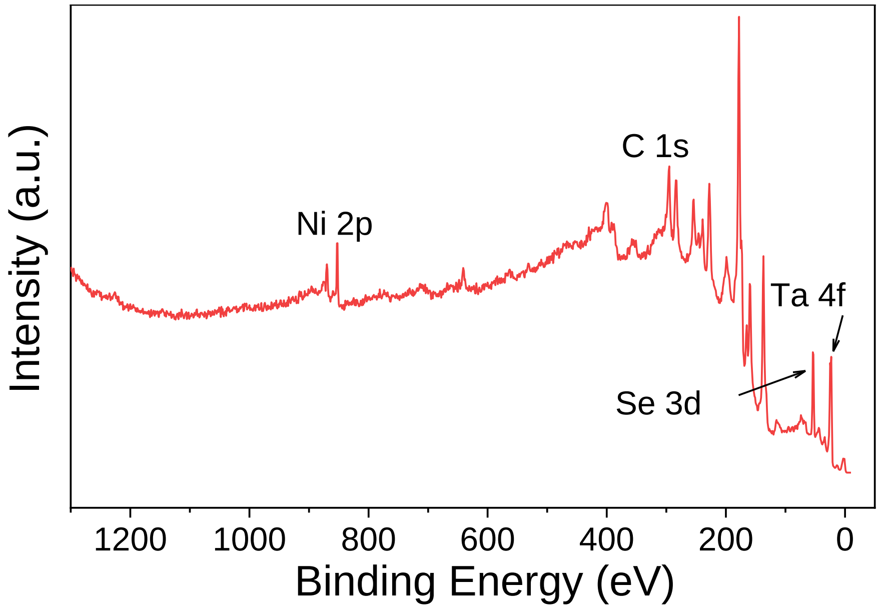

Figure S2. Wide-scan XPS spectrum of the synthesized Ta_2NiSe_5 crystal. It clearly shows the Ta 4f, Ni 2p, and Se 3d signals.

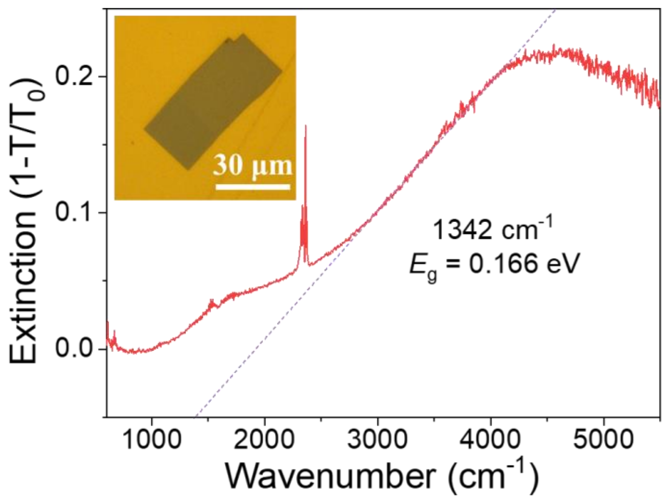

Figure S3. FTIR absorption spectrum of the mechanically exfoliated Ta_2NiSe_5 nanosheets. Insert is the optical microscope image of the Ta_2NiSe_5 nanosheets on the gold substrate. This absorption spectrum shows that the optical band gap of Ta2NiSe$_5$ is ~0.166 eV at room temperature.

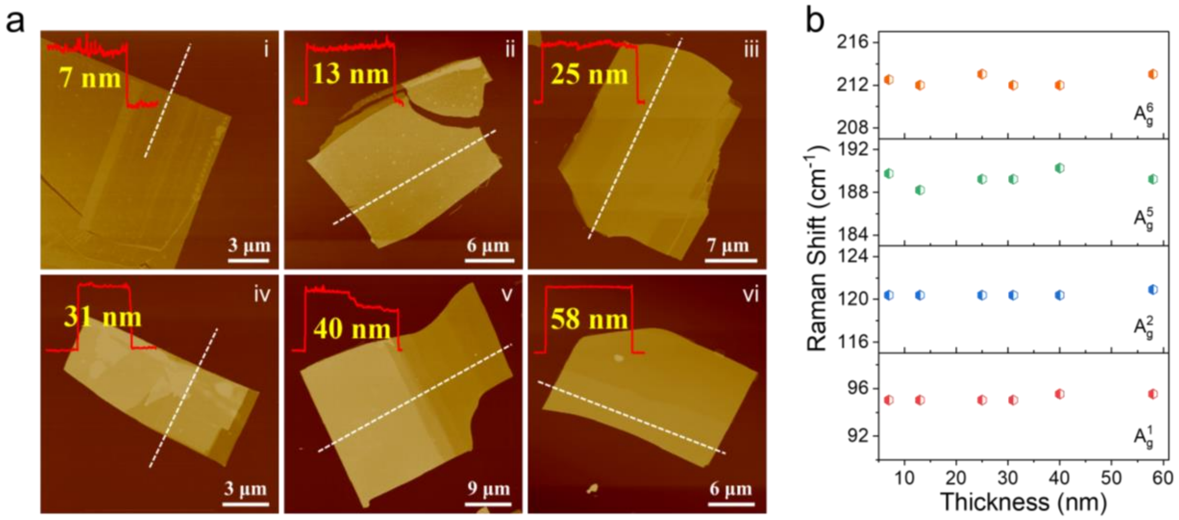

Figure S4. AFM images and Raman spectra of ultrathin Ta_2NiSe_5 crystals at different thicknesses. (a) AFM images of ultrathin Ta_2NiSe_5 nanoflakes at i) 7 nm, ii) 13 nm, iii) 25 nm, iv) 31 nm, v) 40 nm, and vi) 58 nm. (b) Frequencies of Raman modes as a function of the thickness of nanoflakes. All samples show the two strong Raman peaks at ~97 and ~122 cm^{-1}, which are present only in the monoclinic structure. This result can further prove that the EI phase exists in the Ta_2NiSe_5 with different thickness.

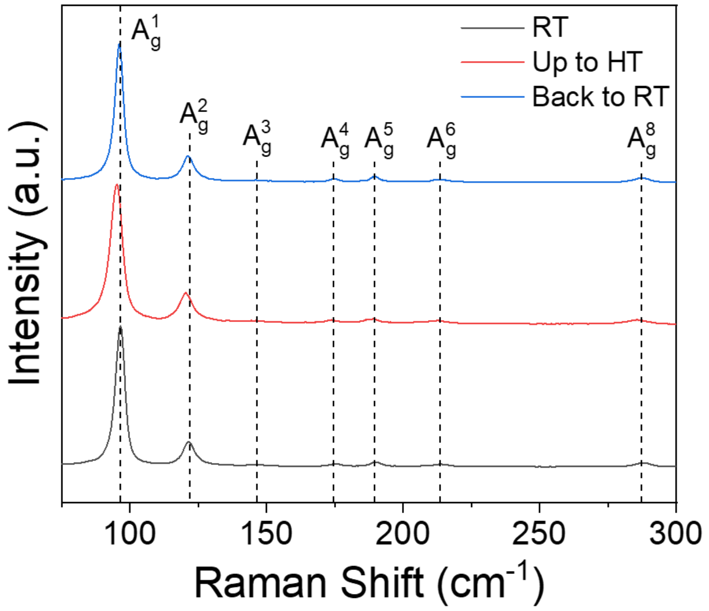

Figure S5. Raman spectra of Ta_2NiSe_5 nanoflake at different temperatures. As the temperature increases, the characteristic peaks have an obvious red shift, which is related to the second-order phase transition of Ta_2NiSe_5.

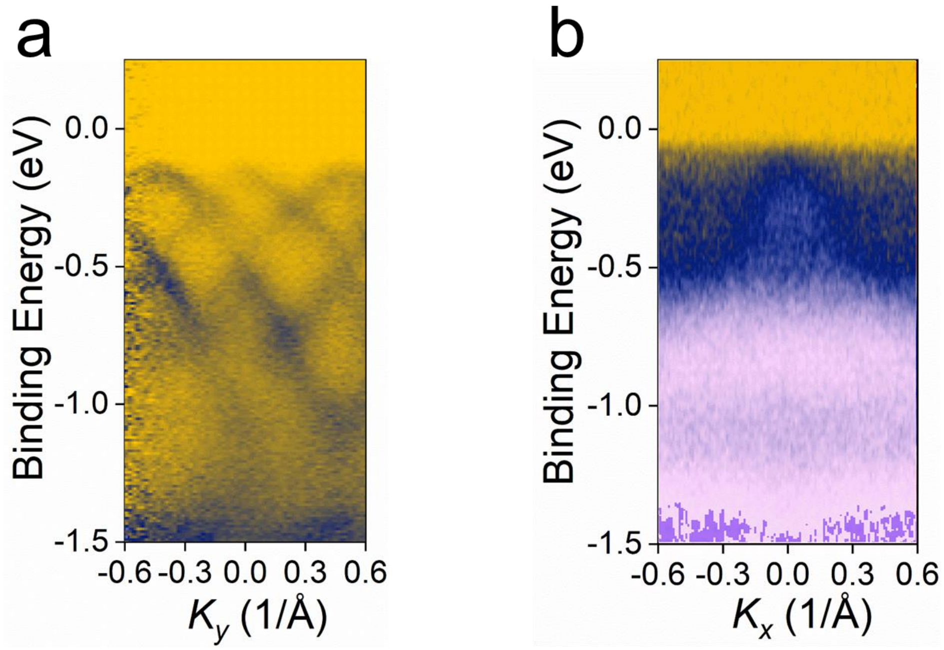

Figure S6. Band structure of the Ta_2NiSe_5 single crystal measured by ARPES at T = 300 K along the $\overline{\Gamma}$-$\overline{\mathrm{Y}}$ direction (a) and at T = 350 K along the $\overline{\Gamma}$-$\overline{\mathrm{X}}$ direction (b). It can be seen that the valence band flattening disappears at T = 350 K.

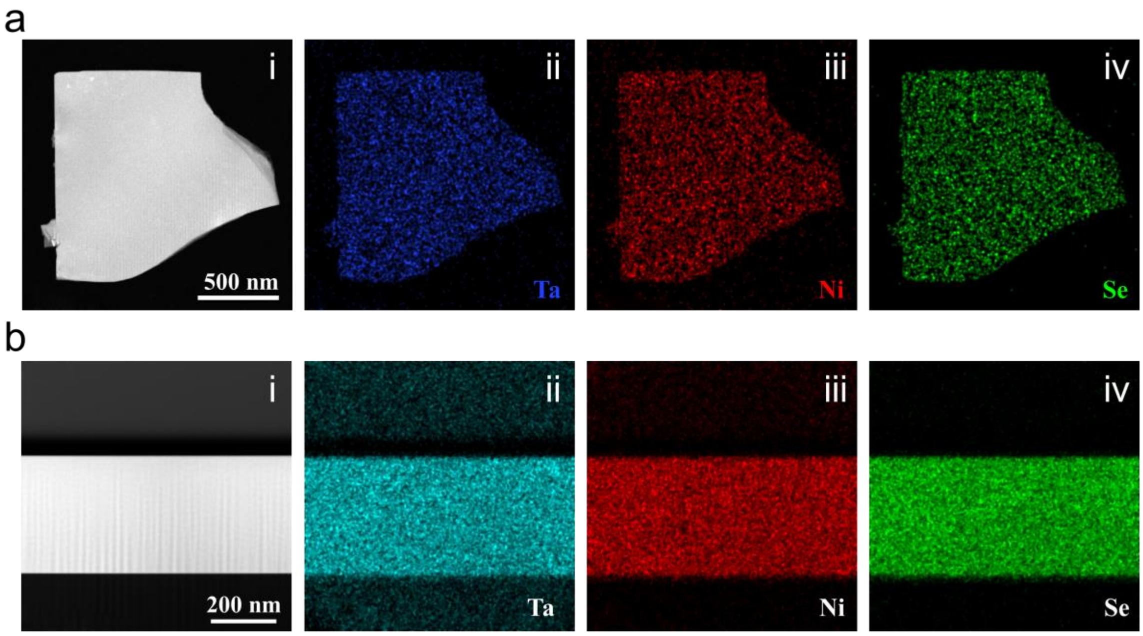

Figure S7. Low-magnification top surface (a) and (b) cross-section HAADF-STEM images of the Ta_2NiSe_5 nanosheet and corresponding EDX element mapping images of Ta, Ni, and Se.

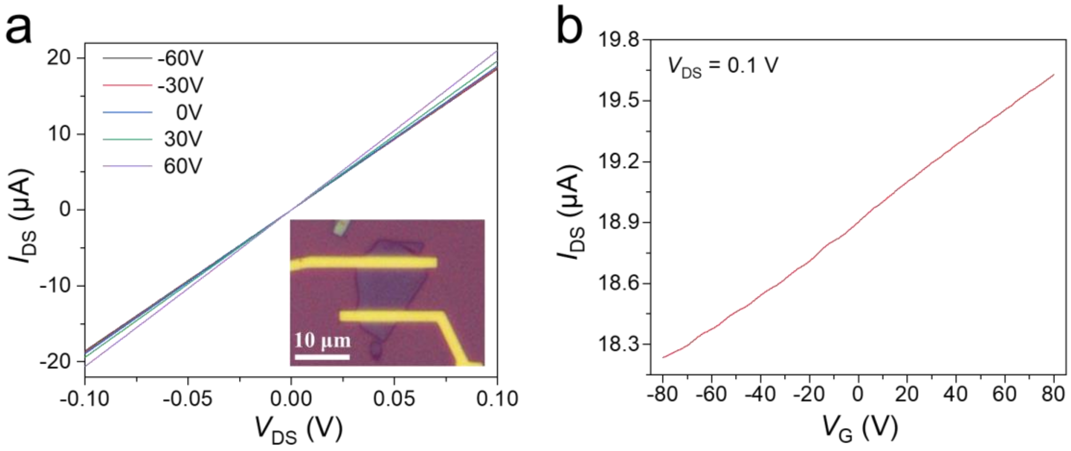

Figure S8. Electrical properties of the FET fabricated with the Ta_2NiSe_5 nanosheet. (a) Output characteristics of Ta_2NiSe_5 FET by sweeping the gate voltage (V_G) from －60 V to 60 V with steps of 30 V. It demonstrates a weak gate-control ability. Inset is the optical microscope image of the Ta_2NiSe_5 FET. (b) Transfer characteristics for the Ta_2NiSe_5 FET with a fixed source-drain bias V_{DS} = 0.1 V. It exhibits typical *n*-type semiconductor characteristics.

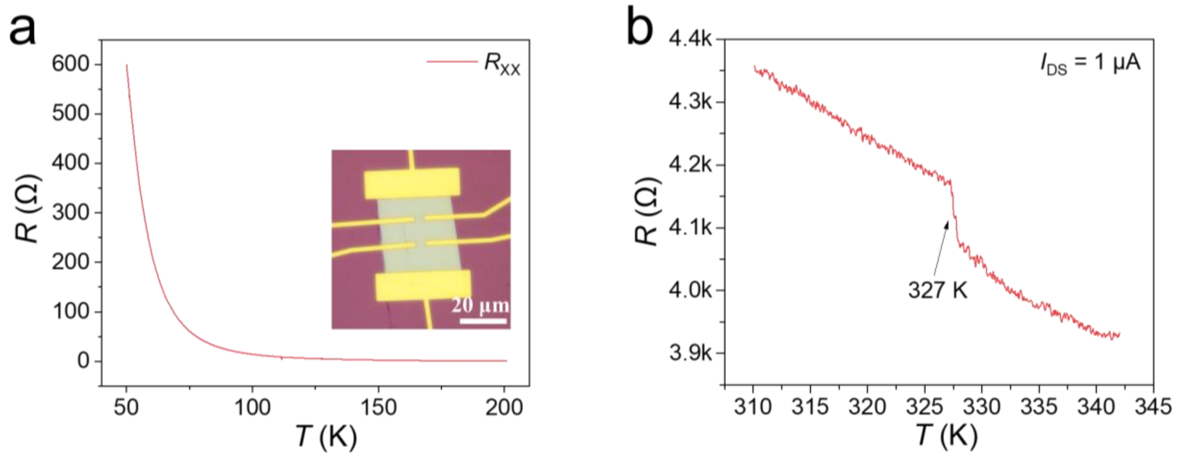

Figure S9. Transport properties of the Ta_2NiSe_5. (a) Temperature dependence of the longitudinal resistance R_{XX} of the Ta_2NiSe_5 nanoflake. Inset is typical optical image of the Ta_2NiSe_5 nanoflake-based Hall device. It can be seen that the resistance increases with the decreasing temperature, showing a typical semiconductor behavior. (b) Temperature dependence of the resistance of the Ta_2NiSe_5 Hall device at fixed source-drain current I_{DS} = 1 μA displays an anomaly at T~327 K, which corresponds to the EI transition.

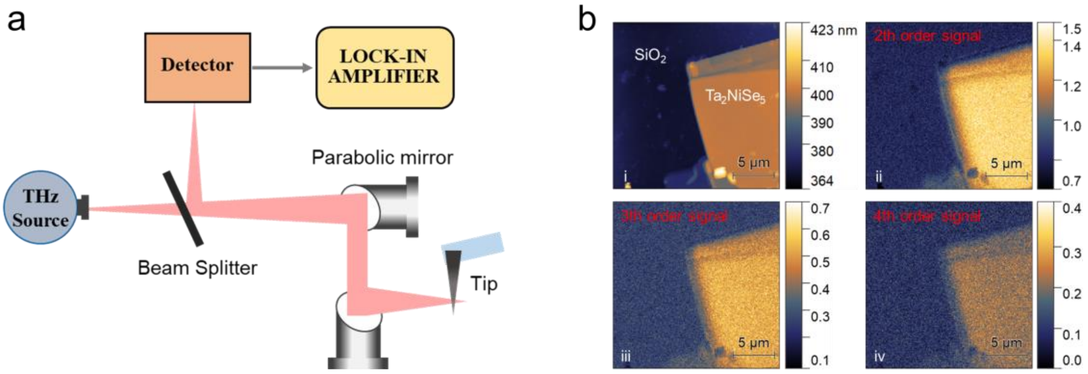

Figure S10. THz s-SNOM test of the Ta_2NiSe_5 sample. (a) Schematic diagram of the THz s-SNOM experimental setup. It consists of THz source, a pair of parabolic mirrors, beam splitter, THz detector, lock-in amplifier, and an AFM tip. (b) AFM topography image (i) and the THz near-field microscopy mapping images (ii-iv) from different orders of the scattered signal (2-4th order signals) of the Ta_2NiSe_5 nanoflake on the Si/SiO_2 substrate. All mapping images show the obvious THz near-field distribution of the Ta_2NiSe_5 nanoflake and considerable optical contrast between Ta_2NiSe_5 and Si/SiO_2 substrate.

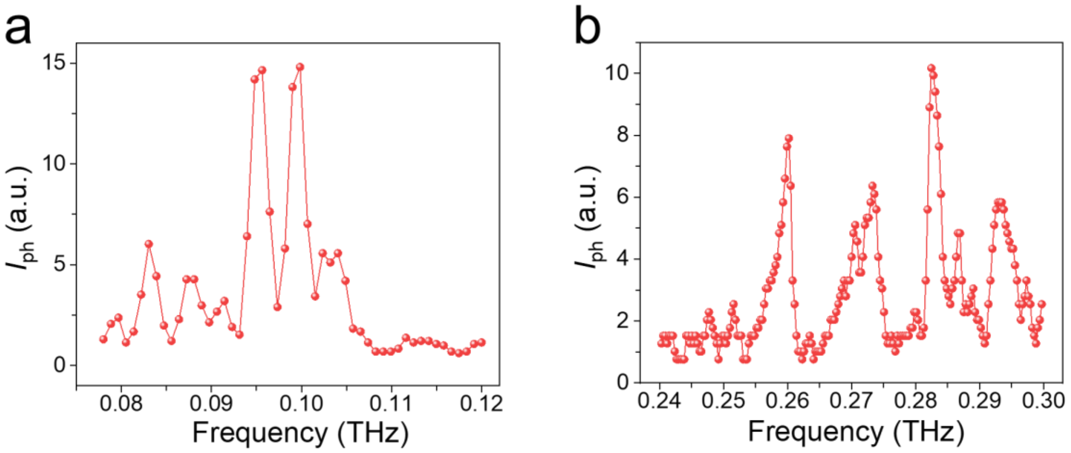

Figure S11. Normalized I_{ph} of the Ta_2NiSe_5 photodetector at a frequency 0.07-0.12 THz (a) and 0.24-0.30 THz (b) at bias voltage of 0.1 V. It can be seen that the photodetector exhibits a prominent photocurrent response in the wavebands of 0.07-0.30 THz at 0.1 V bias voltage.

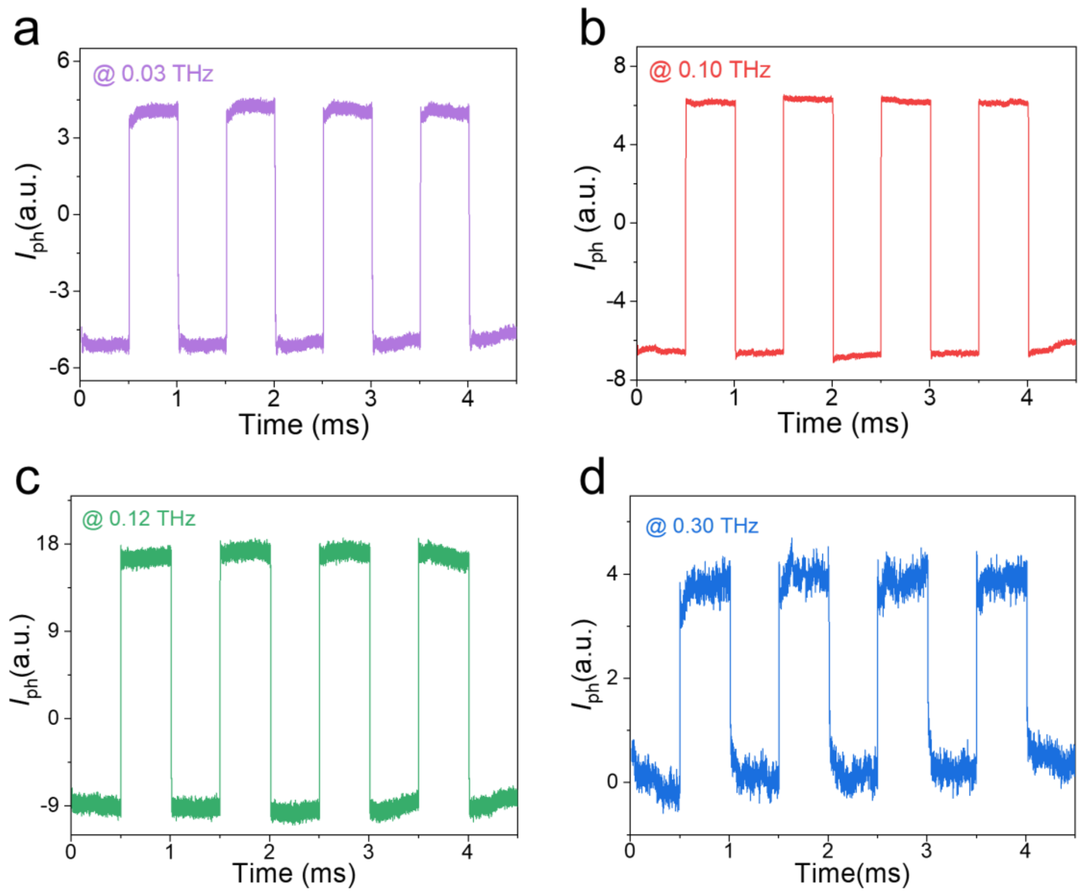

Figure S12. Time-resolved photoresponse spectra of the Ta_2NiSe_5 photodetector irradiated with 0.03 (a), 0.10 (b), 0.12 (c), and 0.30 THz (d) wave at 0.1 V bias voltage. It can be seen that the Ta_2NiSe_5 photodetector displays the fast and stable response waveform at 0.03, 0.10, 0.12, and 0.30 THz, which further illustrates the broadband detection characteristics.

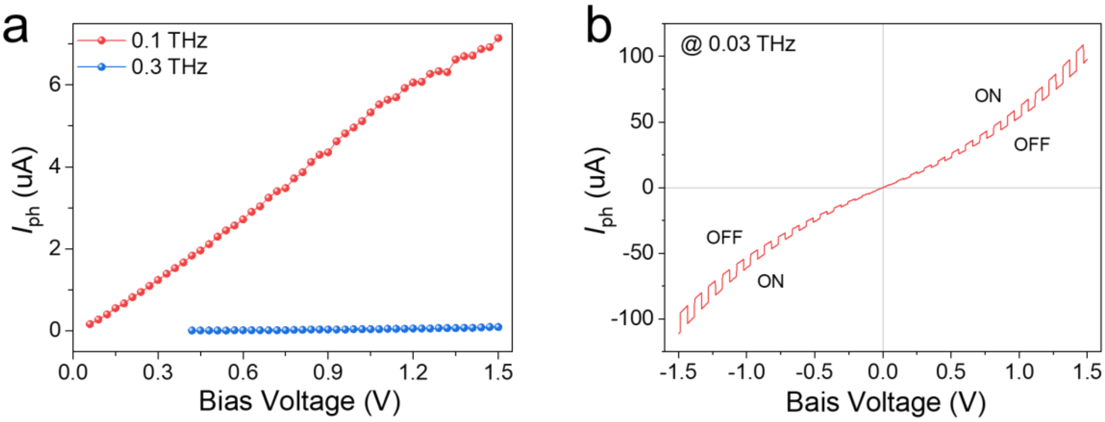

Figure S13. (a) The evolution of *I*-*V* curve for the Ta_2NiSe_5 photodetector under "ON/OFF" modulated radiation at 0.03 THz. The I_{ph} of the photodetector grows with increasing bias voltage, and still maintains a stable response waveform. (b) Bias voltage-dependent Iph of the photodetector irradiated with 0.10 and 0.30 THz wave.

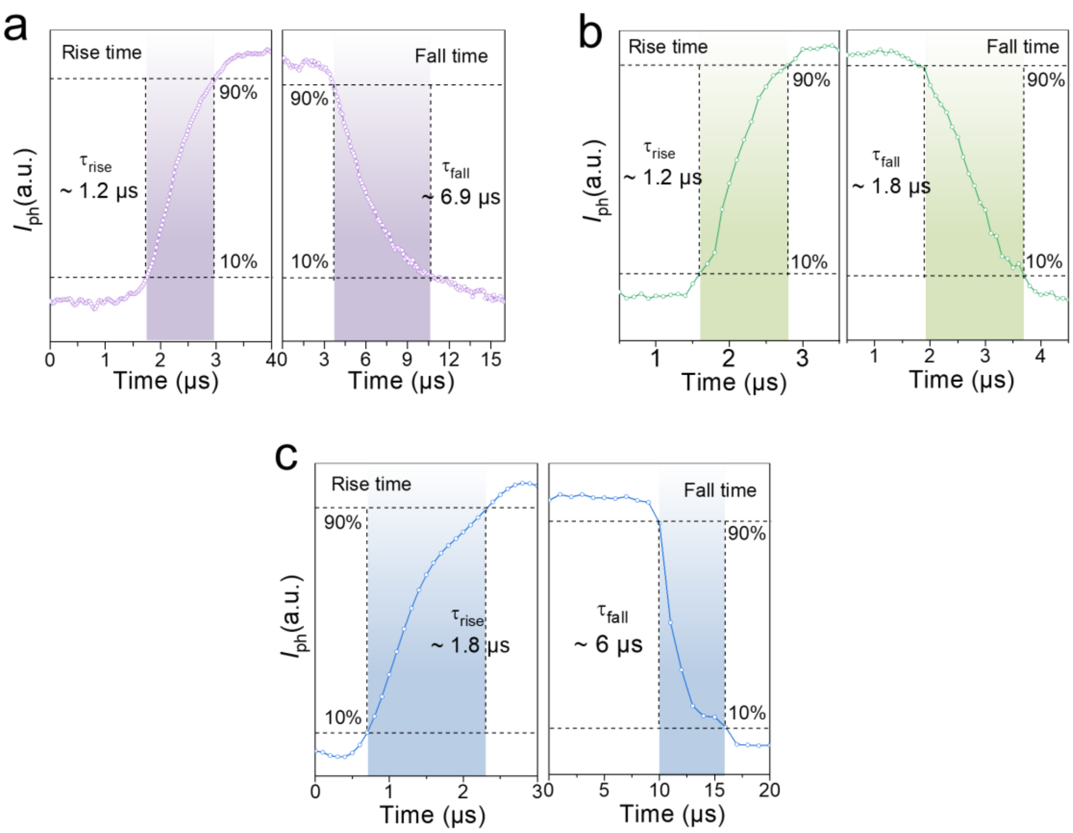

Figure S14. Rising/falling times for the Ta_2NiSe_5 photodetector under 0.03 (a), 0.12 (b), and 0.30 THz (c) illumination at a bias voltage of 0.1 V.
